# Specialist recommendation for chemoprevention medications in patients at familial risk of breast cancer: a cross-sectional survey in England

**DOI:** 10.1007/s12687-020-00490-4

**Published:** 2020-10-28

**Authors:** Siang Ing Lee, Helen Curtis, Sadaf Qureshi, Brittany Dutton, Nadeem Qureshi

**Affiliations:** 1 Division of Primary Care, School of Medicine, University of Nottingham, Tower Building, University Park, Nottingham, NG7 2RD UK; 2 EBM DataLab, Centre for Evidence Based Medicine, Nuffield Department of Primary Care Health Sciences, University of Oxford, Oxford, OX UK; 3 NHS Derby and Derbyshire Clinical Commissioning Group, Derby, UK

**Keywords:** Genetic predisposition to disease, Breast neoplasm, Familial breast cancer, Guidelines

## Abstract

In England, the National Institute for Health and Care Excellence guideline for familial breast cancer recommends chemoprevention for women at high and moderate familial risk of breast cancer. However, prescribing of chemoprevention has not improved since the introduction of the guideline in 2013. The study aims to identify the current practice, in England, of familial cancer specialists offering chemoprevention and recommending prescribing in primary care. This was an anonymized national cross-sectional survey of familial breast cancer risk services in England. Lead clinicians were sent an online survey link. The survey questions included whether chemoprevention was offered/considered for high- and moderate-risk women, when chemoprevention prescribing and recommendation to primary care started, medications prescribed, age groups considered for chemoprevention, and existence of a shared prescribing protocol with primary care. The survey was sent to 115 hospital services; responses from 50 services (43%) were included in the analysis. Of the 40 services offering chemoprevention for high-risk women, 15 (38%) did not prescribe but 31 (78%) recommended prescribing to primary care. Of the 31 services considering chemoprevention for moderate risk, eight (26%) did not prescribe with 26 (84%) recommended prescribing to primary care. Only three services reported having a shared protocol with primary care. Within 3 years of the guidelines, many services recognized the role of chemoprevention for both high and moderate risk with a key role for primary care to initiate prescribing. However, there is still room for improvement.

**Electronic Supplementary Material:**

The online version of this article (10.1007/s12687-020-00490-4) contains supplementary material, which is available to authorized users.

## Introduction

Breast cancer is the most common cancer in women worldwide (World Cancer Research Fund 2019). In developed countries, one in eight women will develop breast cancer in their lifetime (Cancer Research UK 2018). Around 5–10% of breast cancers are caused by an inherited faulty gene such as the *BRCA1* and *BRCA2* genes (Cancer Research UK 2018). Family history of breast and related cancers is recognized as a risk factor and has been used to stratify disease risk (Cancer Research UK 2018; NICE 2013b). For instance, in England, the National Institute for Health and Care Excellence (NICE) categorizes familial breast cancer (FBC) risk into near population risk (lifetime risk < 17% from age 20 and < 3% between ages 40 and 50), moderate risk (lifetime risk of 17–29% from age 20 or 3–8% between ages 40 and 50), and high risk (lifetime risk of ≥ 30% from age 20 or > 8% risk between ages 40 and 50) (NICE 2013b).

At present, FBC is not routinely screened for and women are assessed opportunistically when they present with concerns about their family history (NICE 2013b; Qureshi et al. 2020). Women identified at risk of FBC are usually referred to specialist for further assessment and management of their breast cancer risk. The pathway to specialist care is based on the local protocol. Most commonly all women are referred to specialist family history clinics, with moderate risk assessed and followed up in this setting, while those confirmed to be at high risk referred to clinical genetics for more detailed counseling (NICE 2013b; Smith et al. 2016b). The options for risk reduction measures include increased surveillance, prophylactic surgery, and chemoprevention (NICE 2013b). Chemoprevention can reduce the incidence of breast cancer by over 30% (Cuzick et al. 2013).

In 2013, the English NICE guideline for FBC recommended that women at high risk of FBC be *offered* chemoprevention (tamoxifen or raloxifene). In 2017, the guideline was updated to include anastrozole as chemoprevention for post-menopausal women. For women at moderate risk of FBC, chemoprevention should be *considered* (NICE 2013b). This difference in terminology reflects the strength of the recommendation: the word *offer* is used to reflect a strong recommendation, usually where there is clear evidence of benefit, while the word *consider* is used to reflect a recommendation for which the evidence of benefit is less certain (NICE 2014).

Chemoprevention medications are usually initiated by specialists and primary care may later take over prescribing of chemoprevention for the recommended 5-year course. An observational study using national primary care prescribing data has shown a slow uptake of this updated NICE guideline; assuming 10% of all eligible women would accept the offer of chemoprevention, the study estimated an 83% shortfall against the predicted 50,000 uptake (Curtis et al. 2018).

Anecdotally, this may be due to specialists not recommending treatment to women with moderate risk. Qualitative research found that general practitioners are more willing to continue prescribing chemoprevention if it was initiated in secondary care under a shared care agreement (Rainey et al. 2020; Smith et al. 2017). A further consideration is the current practice of specialist recommending chemoprevention for women at risk of FBC.

This study aims to identify which familial cancer services in England recommend chemoprevention for women at high and moderate risk of familial breast cancer in line with the 2013 NICE guidelines and if they advise primary care to prescribe chemoprevention.

## Methods

### Ethical approval

This study was approved by the Health Research Authority (REC reference: 19/HRA/3946) and the University of Nottingham Faculty of Medicine and Health Sciences Research Ethics Committee (reference: 326-1906). Individual Research and Development offices for all potential NHS trusts (hospital services) in England were sent local information packs and contacted to confirm capacity and capability.

### Identification of familial breast cancer services

Using the NHS directory website, all NHS trusts in England were identified and screened for FBC services (NHS 2019b). Information about the types of services offered was listed on the NHS directory website and the individual trust websites; those that did not indicate or have documentation of offering FBC services were excluded at this initial screening stage. Regional genetics centers were identified from the British Society for Genetic Medicine (BSGM 2019) website.

Following this, initial contact (e-mail or telephone) was made with all relevant departments to further clarify whether FBC services were offered, who the lead clinicians were, and their correct contact details. This was initiated by using the generic contact details that were available publicly. When this was not available, attempts for contacts were made via hospital contact numbers (NHS 2019b).

### Recruitment

Once provision of FBC services and methods to contact the lead clinician was ascertained, information about the research and a link to the online survey were sent via e-mail. In some cases, the secretary or staff would forward this to the lead clinician on behalf of the researcher. Reminders were sent after four weeks.

### Questionnaire

A short questionnaire was designed to obtain the following information:

- Does the service recommend chemoprevention for people at high or moderate risk of FBC? If so, in which year have they started prescribing and recommending the prescribing to primary care?
- Is chemoprevention recommended for a specific age group?
- What chemoprevention medication is recommended?
- Which primary care organizations (called clinical commissioning groups (CCGs)) do the hospital service receive referrals from?
- Is there a local shared care protocol with primary care organization for FBC chemoprevention?
- Characteristics of the service (population size, number of consultations)
- Job role of the survey respondent

Please see Supplementary Material 1 for the questionnaire. The CCGs listed in the questionnaire were up to date as of April 2019 (NHS 2019a; NHS England 2019).

The face validity of the questionnaire was checked with a FBC specialist, a general practitioner, and a health service researcher. The questionnaire was hosted online using the Online Survey and a link was sent to lead clinicians of participating trusts (Online Surveys 2019). One month after the questionnaire was launched, minor changes to the questions’ wording was made based on feedback from participants to align with the wording of the NICE guideline recommendations; specifically the word “recommended” was rephrased to “offered” for high FBC risk and to “considered” for moderate FBC risk.

The questionnaire responses were anonymized. In instances of ambiguity, further clarification of responses was sought if participants had agreed for further contact and provided contact details in the questionnaire.

## Results

### Response rate

**Fig. 1:**
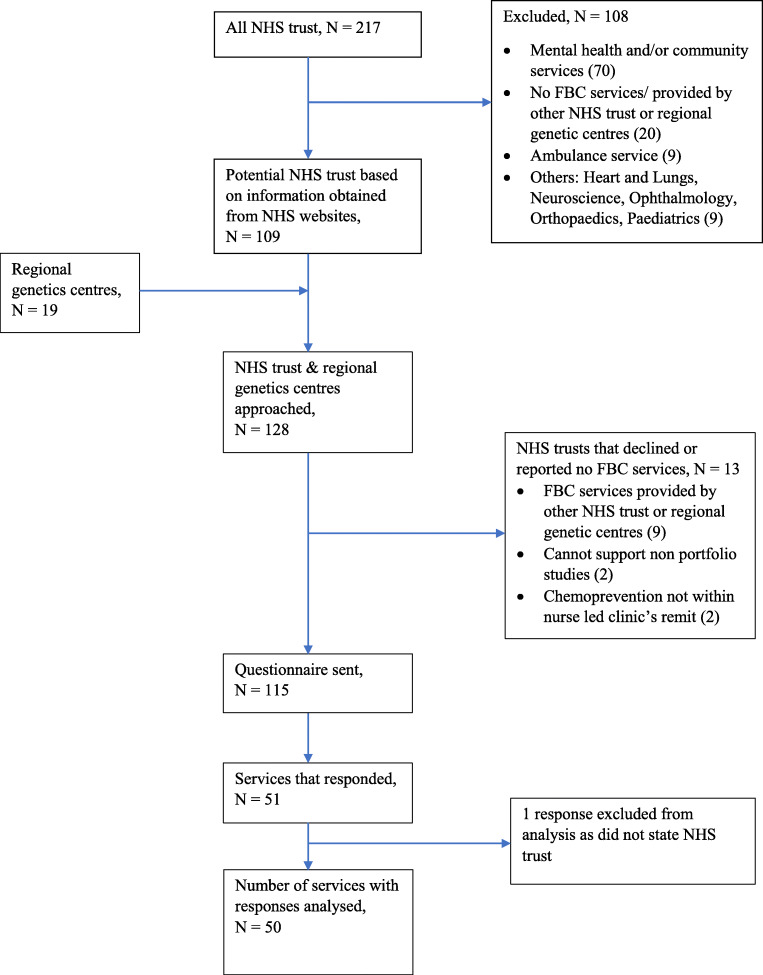
Flowchart of the recruitment for the questionnaire study

Figure 1 outlines the recruitment process. Two hundred and seventeen NHS trusts (hospital services) were identified from the NHS directory as of May 2019, of which 109 trusts were identified as potential sites that provide FBC services based on information from the NHS directory websites. Together with the 19 regional genetics services listed on the BSGM website, initial contact was made to a total of 128 services. Thirteen services declined or reported having no FBC services; the reasons are listed in Fig. 1.

Overall, the questionnaire was sent to 115 services in July–September 2019; 53 responses were received from 51 services. Responses from 50 services (43%) were included for analysis: two FBC services provided two responses each, and these were combined so that each service is represented by one response; one response was excluded from the analysis as it did not state which NHS trust the response was for.

### Characteristics of respondents

Table 1 shows the characteristics of the 52 respondents representing 50 services. Eighty-one percent were from breast/ breast cancer specialty. There were slightly more responses from doctors (*n* = 29) than from nurses (*n* = 21), with 19 doctors identifying themselves as consultants. For the two NHS trusts that had two respondents each, both were breast specialists; in one trust, replies were received from a nurse and a surgeon, while the other trust was from a nurse and a physician.

**Table 1:** Characteristics of the 52 respondents for the 50 services included in the analysis

Characteristics	Number (%)
Specialty, *n* = 52	
Breast	42 (81)
Genetics	10 (19)
Respondents’ health care role, *n* = 52	
Doctor	29 (56)
Physician	20 (38)
Surgeon	9 (17)
Nurse	21 (40)
Others*	2 (4)
Regions of the CCG covered by the service**	
East Midlands	4
Eastern	8
Greater Manchester	4
Kent Surrey Sussex	5
North East and North Cumbria	3
North Thames	4
North West Coast	5
North West London	2
South London	1
South West Peninsula	3
West of England	3
Wessex	3
West Midlands	3
Yorkshire and Humber	7

*Others include clinical manager and radiographer

**Total number adds up to 55 as 6 services covered 2 CCG regions and 1 service did not report its CCG regions

The 64 services that did not respond had similar characteristics to responders: 14% (9/64) of non-responders were genetic services, with remaining sent to breast services. Considering the health professionals sent the survey, 52% (31/60) of non-responders were doctors, 40% (24/60) were nurses, and 8% (5/60) were other professionals.

### Characteristics of services

**Fig. 2:**
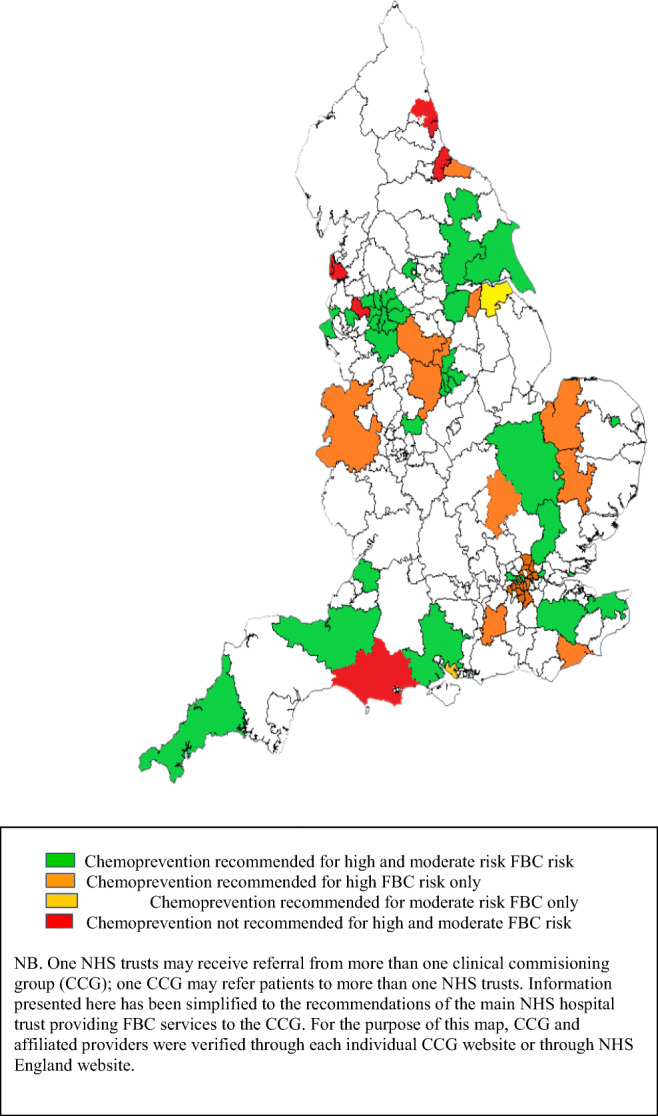
Map of reported specialist recommendation for chemoprevention across English primary care organizations (clinical commissioning groups)

There was a good geographical spread of responses, as indicated by primary care organizations (CCGs) covered by respective familial breast cancer services (Table 1; Fig. 2). Thirty-nine services reported the size of the population they served; this ranged from 14,000 to 5,500,000, with a median of 700,000 and interquartile range of 307,500 to 2,400,000. Three services reported also receiving out of area referral and therefore could not quantify the population size they served.

Forty-four services reported the number of consultations conducted annually for familial breast cancer risk assessment; this ranged from 50 to 3000, with a median of 237 and interquartile range of 150 to 525. Two FBC services reported that the risk assessments were conducted by affiliated genetic services. Four services reported that low-risk patients were not seen in clinic.

### Chemoprevention recommendation by specialist

Overall, 42 of the 50 NHS hospital services provided chemoprevention for women at high and/or moderate risk of FBC. Using verified information from individual primary care organization (CCG) websites or through NHS England websites, the 42 services were affiliated to 45 CCGs, covering a total population of 15,840,484. Looking in more detail, 80% (40/50) of services offered chemoprevention for high FBC risk, while 63% (31/49) of services considered chemoprevention for moderate FBC risk (Table 2). The map in Fig. 2 demonstrates the wide variation in chemoprevention recommendations, even between adjacent primary care organizations.

**Table 2:** Chemoprevention recommendation by specialist for women at high or moderate risk of familial breast cancer (FBC)

	Recommendation of chemoprevention by participating services, *n* (%)	
	Yes	No
By FBC risk		
High risk, *n* = 50	40 (80)	10 (20)
Moderate, *n* = 49	31 (63)	18 (37)
Combination of responses, *n* = 50	*N* (%)	
Yes for high and moderate risk	29 (58)	
Yes for high risk, no for moderate risk	11 (22)	
No for high risk, yes for moderate risk	2 (4)	
No for high and moderate risk	7 (14)	
No for high, no entry for moderate risk	1 (2)	
High risk, *n* = 50		
Responses by specialty		
Breast	31 (78)	9 (23)
Genetics	9 (90)	1 (10)
Responses by health care role		
Doctor	26 (90)	3 (10)
Physician	18 (90)	2 (10)
Surgeon	8 (89)	1 (11)
Nurse	13 (68)	6 (32)
Others*	1 (50)	1 (50)
Moderate risk, *n* = 49		
Responses by specialty		
Breast	23 (59)	16 (41)
Genetics	8 (80)	2 (20)
Responses by health care role		
Doctor	20 (69)	9 (31)
Physician	14 (70)	6 (30)
Surgeon	6 (67)	3 (33)
Nurse	11 (61)	7 (39)
Others*	0	2 (100)

*Others include clinical manager and radiographer

When the responses were grouped by the respondent’s specialty, the proportion of respondent who would recommend chemoprevention was higher for genetic specialists than for breast specialists. Although a high proportion (78%, 31/40) of breast specialists would offer chemoprevention for high FBC risk, this dropped to 59% (23/39) for moderate FBC risk. In comparison, the drop was marginal for genetic specialists from 90 (9/10, high FBC risk) to 80% (8/10, moderate FBC risk).

When the responses were grouped by the respondent’s health care role, there was a higher proportion of doctors (90%, 26/29) that would recommend chemoprevention compared to nurses (68%, 13/19) for high FBC risk, but the proportion was similar between doctors (69%, 20/29) and nurses (61%, 11/18) for moderate FBC risk. The related formal statistical comparison is provided in Supplementary Material 2.

Fifty-eight percent (29/50) of services recommended chemoprevention for both high and moderate FBC risk; 22% (11/50) recommended chemoprevention for high FBC risk but not moderate risk. For the two responses where chemoprevention was recommended for moderate FBC risk but not high FBC risk, both were nurses from breast services. It was not possible to contact respondents to clarify responses. For the seven services that did not recommend chemoprevention for both high and moderate FBC risk, three respondents were doctors, three were nurses, and one was a radiographer; six were breast specialists and one was a genetic specialist.

All services that did recommend chemoprevention indicated use of tamoxifen. Fewer services discussed the use of anastrozole or raloxifene (Table 3).

**Table 3:** Chemoprevention medication for familial breast cancer risk

**Familial breast cancer chemoprevention medication**	**Number of services that offer/consider the medication for chemoprevention**	
	**High risk,** ***n*** **= 40**	**Moderate risk,** ***n*** **= 31**
Tamoxifen	37	30
Anastrozole	23	19
Raloxifene	21	20
No entry	3	1
**Is chemoprevention offered to specific age group**	**Number of services**	
	**High risk,** ***n*** **= 40**	**Moderate risk,** ***n*** **= 31**
Yes	27	19
No	11	10
Not sure	–	1
No entry	2	1
**Which age group (years)**	**High risk,** ***n*** **= 27**	**Moderate risk,** ***n*** **= 19**
> 30	–	1
≥ 35	16	10
> 40	1	2
> 45	1	–
40–50	1	2
40–60	1	1
Ensure completed family if child-bearing age	2	2
No valid entry	5	1

### Chemoprevention for specific age groups

Most services recommended chemoprevention for women aged 35 and over. Generally, if services recommended chemoprevention for both high- and moderate-risk women, they report the same age group for high and moderate risk. Two services reported they would ensure that women of child-bearing age had completed their family before chemoprevention was recommended (Table 3).

### Initiation of chemoprevention in each hospital service

i. High risk

Seventeen (43%) of the services offering chemoprevention for high-risk women started prescribing chemoprevention before or within three years of NICE recommending chemoprevention (2013). Eight (20%) took more than three years to adopt the guidelines.

The remaining 15 services discussed chemoprevention for high-risk women but did not prescribe the medication themselves, with six services reporting other colleagues prescribed the chemoprevention (in five services, the other colleagues were General Practitioners). Of the nine genetic specialists that would offer chemoprevention, two (22%) indicated the genetic service prescribed chemoprevention, compared to 74% (23/31) of breast specialists.

From the free-text comments, one service commented that chemoprevention was offered as a bridge until risk reduction surgery for high-risk patients; two services commented that high-risk patients were more likely to choose risk reduction surgery (Table 4).

ii. Moderate risk

**Table 4:** Years when services started prescribing/recommending chemoprevention to primary care and when shared care protocols were written

	Number of services
Year when services started prescribing chemoprevention	
High risk, *n* = 40	
Before 2013	2
2013	4
1–3 years after the 2013 NICE guideline	11
> 3 years after the 2013 NICE guideline	8
Responding service offers/discusses but does not prescribe*	15
General practitioner prescribes	5
Oncologist prescribes	1
Secondary care prescribes	1
Do not prescribe/not applicable	9
Moderate risk, *n* = 31	
Before 2013	2
2013	5
1–3 years after the 2013 NICE guideline	8
> 3 years after the 2013 NICE guideline	7
Year not specified	1
Responding service considers/discusses but does not prescribe*	8
General practitioner prescribes	2
Oncologist prescribes	1
Secondary care prescribes	1
Do not prescribe/not applicable	5
Year when services started recommending chemoprevention to primary care	
High risk, *n* = 40	
Before 2013	1
2013	7
1–3 years after the 2013 NICE guideline	14
> 3 years after the 2013 NICE guideline	9
Do not know	3
Recommends to secondary care	1
No direct recommendation but expect primary care to continue prescribing	1
Chemoprevention medication not prescribed in primary care	1
Prescribes but does not recommend to primary care	1
Decision by general practitioners	1
Management by breast team	1
Moderate risk, *n* = 31	
Before 2013	1
2013	7
1–3 years after the 2013 NICE guideline	9
> 3 years after the 2013 NICE guideline	8
Year not specified	1
Management by local breast teams	1
Recommends to secondary care	1
Chemoprevention medication not prescribed in primary care	1
Expects that GP will continue	1
Missing	1
Written shared care protocol for prescribing chemoprevention in primary care with the local CCG, *N* = 50	
Yes	3
No	30
Not sure	17

*Total number in the table and in the main text may not add up as more than one options provided

Fifteen (48%) of the services considering chemoprevention for moderate-risk women started prescribing chemoprevention before or within three years of NICE recommending chemoprevention (2013). Seven (23%) services took more than three years to adopt the guidelines.

The remaining eight services discussed chemoprevention for moderate-risk women but did not prescribe the medication themselves, with three services reporting other colleagues prescribed the chemoprevention (Table 4). Of the eight genetic specialists that would consider chemoprevention, three (38%) indicated the genetic service prescribed chemoprevention, compared to 87% (20/23) of breast specialists.

### Prescribing of chemoprevention in primary care

As indicated in Table 4, before or within three years of NICE recommending chemoprevention, around half of services that recommended chemoprevention indicated that primary care should prescribe this medication. When specifically considering high-risk women, specialist services were more likely to recommended that primary care prescribe chemoprevention than initiate the medication themselves.

The recommendation for primary care to prescribe and specialist prescribing was similar for moderate risk (within three years 17 vs 15 services, any time 26 vs 23 services). Five services did not make the recommendation to primary care for high FBC risks with reasons listed in Table 4.

### Shared care protocol with primary care

Only three services reported having a shared care protocol for prescribing chemoprevention with the local primary care organization (CCG). A third of the respondents were not sure if there was a shared care protocol (Table 4).

## Discussion

### Main findings

The majority of hospital services recommended chemoprevention medication for women at high familial risk of breast cancer, with smaller proportions recommending chemoprevention for moderate-risk women. Of these, around half started prescribing and/or recommending primary care clinicians to prescribe chemoprevention within three years of the introduction of NICE guideline, in 2013. Furthermore, it was more likely that primary care would be recommended to prescribe than specialist services start the medication themselves.

### Relationship to current literature

The higher proportion of high-risk patients recommended chemoprevention is consistent with strength of evidence for benefits of chemoprevention (Fisher et al. 2005; NICE 2013a). As well as concern about the evidence base, reluctance to prescribe chemoprevention may be related to concerns of the side-effect profile (including thromboembolism and endometrial cancer) and that chemoprevention was not originally licensed for primary prevention of breast cancer in the UK (Cuzick et al. 2013; NICE 2013b; Smith et al. 2017). Currently, only tamoxifen is licensed for primary prevention of breast cancer in women at moderate to high risk in the UK and this was only recently licensed in 2018 (MHRA 2018).

A recent qualitative study of general practitioners and familial breast cancer specialists identified the barriers of implementing chemoprevention for FBC prevention in the UK (Smith et al. 2016b). Some of the reasons quoted include lack of perceived benefit and being poorly informed of the chemoprevention. Furthermore, a focus group of family cancer clinicians in Australia recognized similar barriers (Keogh et al. 2009).

Smith et al. (2016b) also identified the lack of clarity in the NICE guideline as to who should be initiating the prescription and offer subsequent patient care. The lack of clarity on the most appropriate physician for prevention advice was also one of the top five reasons for reluctance to prescribe tamoxifen or raloxifene for FBC prevention reported in a survey of European breast cancer specialists in 2018 (Noonan et al. 2018). The limited free-text comments from this study also showed that the role of initiating and continuing chemoprevention prescribing varied according to local arrangements.

Even if chemoprevention was offered by clinicians, the uptake among women at increased risk of breast cancer was low (16%) (Smith et al. 2016a). This highlights the importance of efforts to ensure that chemoprevention is being recommended and clinicians and women are supported to make the decision that is right for the individual woman.

A few services commented that high-risk patients prefer risk-reducing surgery over chemoprevention. This is similar to previous literature where high-risk women are more likely to choose prophylactic surgery (Evans et al. 2001). Observational studies have shown that prophylactic surgery reduced the risk of breast cancer by 90% and breast cancer deaths by 81–94% (Hartmann et al. 1999). In contrast, randomized controlled trial reported that tamoxifen reduced breast cancer risk by 35% and there is a lack of evidence on its effect on mortality (Cuzick et al. 2007; Fisher et al. 2005; NICE 2013a).

### Strength

To our knowledge, this is the first study to ascertain the adoption of chemoprevention by specialist in England since the introduction of the NICE guidelines. A comprehensive recruitment strategy was employed nationally to get a sample as representative as possible. The respondents comprised of a range of health care professionals and specialty and covered a wide geographical area.

### Limitation

Although there was a low response rate and risk of responder bias limiting the generalizability of the results, the findings are still useful as it highlights room for improvement in implementing the NICE guideline. Formal comparative analysis between subgroups of specialists was difficult to interpret due to small sample size. Furthermore, it was difficult to identify the lead/senior clinician for each service and those that responded may be completing the survey on behalf of lead/senior clinicians. As a result, respondents may not be able to answer comprehensively the survey questions related to clinical management (such as shared care protocol with primary care).

Identifying the hospital service provision for each primary care organization was challenging as general practices within these organizations may refer to different hospital services. To reduce complexity of the presentation, the information extracted in Fig. 2 was reduced to the most comprehensive service offered for each primary care organization.

### Care pathways and clinical implications

The study demonstrates majority of hospital services have recommended chemoprevention medication for most eligible women aged over 35, in most cases initiating the medication themselves. As well as providing information on implementing chemoprevention recommendations, the survey has given a picture about the workload and configuration of the familial cancer service across England. A few services indicated a triage process in referral pathway with initial cancer risk assessment prior to clinic attendance, for example, using a self-reported family history questionnaire. The multidisciplinary nature of this assessment is demonstrated in this survey by the breadth of respondents.

In the management pathway, it appears that most women were initially seen by breast specialist. Among services that would recommend chemoprevention to at-risk women, a larger proportion of breast specialists prescribed chemoprevention than genetic specialists. This may reflect the longer term management of women at risk of FBC is through the breast specialist team, while the clinical genetics team takes on more of an advisory role with no prescribing responsibilities. Although the survey was completed by several health professionals, the doctors appeared to have lead roles in deciding on chemoprevention. It also appears that most of the hospital initiated prescribing of chemoprevention and delegated the continuation to primary care but very few respondents mentioned formal shared care prescribing protocols between specialist and primary care.

Smith et al. have suggested this could comprise a pro forma issued by specialists when asking general practitioners to prescribe chemoprevention (Smith et al. 2016b). Similarly, Rainey et al. reported that British women indicated a need for protocols to standardize interaction between primary care and hospital specialists to avoid variation in care (Rainey et al. 2020).

Of similar concern, there is question as to whether there is variation in clinical practice across the country. To help address the gap in implementing the NICE FBC chemoprevention recommendations, guideline implementation tools, such as those produced by NICE, may be refined and adopted.

### Future research and policy recommendations

The study highlights a need for clearer shared care protocol between specialist and primary care for the prescribing of chemoprevention. This would improve accountability for and compliance with chemoprevention prescribing.

One challenge of evaluating FBC services was the lack of information on how the FBC services are configured nationally. Referral pathway also differed across the country. This may be addressed by a study that maps out the FBC service pathways in England, for instance who conducts the risk assessments, who discuss chemoprevention with patients, and who does the actual prescribing and monitoring of chemoprevention. This could be facilitated by a centralized record of all FBC services with details of key clinicians in each service. The breast cancer charities may have a role to collate this information.

## Electronic supplementary material

ESM 1 (DOCX 119 kb)

ESM 2 (DOCX 22 kb)
