# Supplementary material for: Specialist recommendation for chemoprevention medications in patients at familial risk of breast cancer: a cross-sectional survey in England
Source: J Community Genet. 2020 Oct 28;12(1):111–20. doi: 10.1007/s12687-020-00490-4 (PMC7846641; doi:10.1007/s12687-020-00490-4)
Supplement: Supplementary file 1 — (DOCX 119 kb) [file 12687_2020_490_MOESM1_ESM.docx]

**Supplementary Material 1: Questionnaire**

**Survey questionnaire vs 1.2**


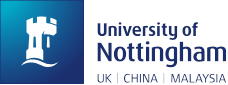

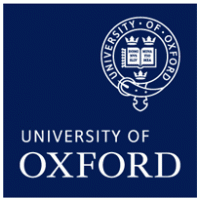


**Survey of Familial Cancer Services on Chemoprevention Recommendations for Familial Breast Cancer (FBC)**

We would like to find out whether your service recommends chemoprevention (e.g. tamoxifen) for people with familial breast cancer risk. We will then map the response to routinely available primary care prescribing data. This will help us understand whether the pattern of prescribing chemoprevention in primary care is associated with specialist recommendations.

**We would be most grateful if you could answer these questions, it should not take any longer than 10 minutes to complete.**

| **Q1. What is your job title?** |
| --- |
|  |
| **Q2. What is the name and locality of your familial cancer specialist service?** |
|  |
| **Q3. Approximately how many consultations does your service perform each year for familial breast cancer risk assessment?** |
|  |
| **Q4. What is the size of the population covered by your familial breast cancer risk assessment service?** |
|  |
| **Q5. Does your service offer chemoprevention for people at *HIGH* risk of FBC, if there is no contraindication?**  *(high risk: ≥30% life time risk OR >8% risk between ages 40 and 50 OR known genetic mutation)* |

- No, *proceed to* ***Q6***
- Yes, *please complete the following box*

| **If answered yes to question 5, please complete the questions below** |
| --- |

**Q5a.** Which year did your service start prescribing chemoprevention?.............................................

**Q5b.** Which year did your service start recommending primary care to prescribe chemoprevention?.....

**Q5c.** Is chemoprevention offered to a specific age group?

- No
- Not sure
- Yes, *please specify:…………………………………………………………………………*

**Q5d** Which chemoprevention medication does your service offer? (*Please tick as many as applicable)*

- Raloxifene
- Other

Please specify…………………………………..

- Tamoxifen
- Anastrozole

| **End of question 5** |
| --- |

| **Q6. Does your service consider chemoprevention for people at *MODERATE* risk of FBC, if there is no contraindication?**  *(moderate risk: >17% to <30% life time risk OR 3 to 8% risk between ages 40 and 50)* |
| --- |

- No, *proceed to* ***Q7***
- Yes, *please complete the following box*

| **If answered yes to question 6, please complete the questions below** |
| --- |

**Q6a.** Which year did your service start prescribing chemoprevention?...............................................

**Q6b.** Which year did your service start recommending primary care to prescribe chemoprevention?.....

**Q6c.** Is chemoprevention considered for a specific age group?

- No
- Not sure
- Yes, *please specify:…………………………………………………………………………*

**Q6d.** Which chemoprevention medication does your service consider? (*Please tick as many as applicable)*

- Raloxifene
- Other

Please specify…………………………………..

- Tamoxifen
- Anastrozole

| **End of question 6** |
| --- |
|  |
| **Q7**. **Has your service written a shared protocol for prescribing chemoprevention in primary care with the local CCG?** |

- No, *proceed to* ***Q8***
- Not sure, *proceed to* ***Q8***
- Yes, *please complete the following box*

| **If answered yes to question 7, please complete the questions below** |
| --- |

**Q7a.** How long did it take to get an agreement with the CCG?.................(weeks)

**Q7b.** Which year did the shared protocol start? ……………….

**Q7c.** I am happy to attach a copy of the shared protocol with this survey / email to the research team at [sianging.lee@nhs.net](mailto:sianging.lee@nhs.net)

- - Yes
  - No

| **End of question 7** |
| --- |

| **Q8. Which CCGs does your service support / receive referrals from?** *(please tick as many as applicable)* | | |
| --- | --- | --- |
| **East Midlands**   - NHS Corby CCG - NHS Derby and Derbyshire CCG - NHS East Leicestershire and Rutland CCG - NHS Leicester City CCG - NHS Lincolnshire East CCG - NHS Lincolnshire West CCG - NHS Mansfield and Ashfield CCG - NHS Nene CCG - NHS Newark and Sherwood CCG - NHS Nottingham City CCG - NHS Nottingham North and East CCG - NHS Nottingham West CCG - NHS Rushcliffe CCG - NHS South Lincolnshire CCG - NHS South West Lincolnshire CCG - NHS West Leicestershire CCG   **West of England**   - NHS Bath and North East Somerset CCG - NHS Bristol, North Somerset and South Gloucestershire CCG - NHS Gloucestershire CCG - NHS Swindon CCG - NHS Wiltshire CCG | **Kent, Surrey and Sussex**   - NHS Ashford CCG - NHS Brighton and Hove CCG - NHS Canterbury and Coastal CCG - NHS Coastal West Sussex CCG - NHS Crawley CCG - NHS Dartford, Gravesham and Swanley CCG - NHS East Surrey CCG - NHS Eastbourne, Hailsham and Seaford CCG - NHS Guildford and Waverley CCG - NHS Hastings and Rother CCG - NHS High Weald Lewes Havens CCG - NHS Horsham and Mid Sussex CCG - NHS Medway CCG - NHS North West Surrey CCG - NHS South Kent Coast CCG - NHS Surrey Downs CCG - NHS Surrey Heath CCG - NHS Swale CCG - NHS Thanet CCG - NHS West Kent CCG | |
| **Thames Valley and South Midlands**   - NHS Berkshire West CCG - NHS Buckinghamshire CCG - NHS East Berkshire CCG - NHS Milton Keynes CCG - NHS Oxfordshire CCG | **South West Peninsula**   - NHS Devon CCG - NHS Kernow CCG - NHS Somerset CCG | |
| **North West Coast**   - NHS Blackburn with Darwen CCG - NHS Blackpool CCG - NHS Chorley and South Ribble CCG - NHS East Lancashire CCG - NHS Fylde and Wyre CCG - NHS Greater Preston CCG - NHS Halton CCG - NHS Knowsley CCG - NHS Liverpool CCG - NHS Morecambe Bay CCG - NHS South Cheshire CCG - NHS South Sefton CCG - NHS Southport and Formby CCG - NHS St Helens CCG - NHS Vale Royal CCG - NHS Warrington CCG - NHS West Cheshire CCG - NHS West Lancashire CCG - NHS Wirral CCG | **North Thames**   - NHS Barking and Dagenham CCG - NHS Barnet CCG - NHS Basildon and Brentwood CCG - NHS Camden CCG - NHS Castle Point and Rochford CCG - NHS City and Hackney CCG - NHS Enfield CCG - NHS Haringey CCG - NHS Havering CCG - NHS Herts Valleys CCG - NHS Islington CCG - NHS Luton CCG - NHS Mid Essex CCG - NHS Newham CCG - NHS Redbridge CCG - NHS Southend CCG - NHS Thurrock CCG - NHS Tower Hamlets CCG - NHS Waltham Forest CCG - NHS West Essex CCG | |
| **Eastern**   - NHS Bedfordshire CCG - NHS Cambridgeshire and Peterborough CCG - NHS East and North Hertfordshire CCG - NHS Great Yarmouth and Waveney CCG - NHS Ipswich and East Suffolk CCG - NHS North East Essex CCG - NHS North Norfolk CCG - NHS Norwich CCG - NHS South Norfolk CCG - NHS West Norfolk CCG - NHS West Suffolk CCG | **Greater Manchester**   - NHS Bolton CCG - NHS Bury CCG - NHS Eastern Cheshire CCG - NHS Heywood, Middleton and Rochdale CCG - NHS Manchester CCG - NHS Oldham CCG - NHS Salford CCG - NHS Stockport CCG - NHS Tameside and Glossop CCG - NHS Trafford CCG - NHS Wigan Borough CCG | |
| South London  - NHS Bexley CCG - NHS Bromley CCG - NHS Croydon CCG - NHS Greenwich CCG - NHS Kingston CCG - NHS Lambeth CCG - NHS Lewisham CCG - NHS Merton CCG - NHS Richmond CCG - NHS Southwark CCG - NHS Sutton CCG - NHS Wandsworth CCG | **North East and North Cumbria**   - NHS Darlington CCG - NHS Durham Dales, Easington and Sedgefield CCG - NHS Hartlepool and Stockton-On-Tees CCG - NHS Newcastle Gateshead CCG - NHS North Cumbria CCG - NHS North Durham CCG - NHS North Tyneside CCG - NHS Northumberland CCG - NHS South Tees CCG - NHS South Tyneside CCG - NHS Sunderland CCG | |
| **North West London**   - NHS Brent CCG - NHS Central London (Westminster) CCG - NHS Ealing CCG - NHS Hammersmith and Fulham CCG - NHS Harrow CCG - NHS Hillingdon CCG - NHS Hounslow CCG - NHS West London CCG | **Wessex**   - NHS Dorset CCG - NHS Fareham and Gosport CCG - NHS Isle of Wight CCG - NHS North East Hampshire and Farnham CCG - NHS North Hampshire CCG - NHS Portsmouth CCG - NHS South Eastern Hampshire CCG - NHS Southampton CCG - NHS West Hampshire CCG | |
| **Yorkshire and Humber**   - NHS Airedale, Wharfedale and Craven CCG - NHS Barnsley CCG - NHS Bassetlaw CCG - NHS Bradford City CCG - NHS Bradford Districts CCG - NHS Calderdale CCG - NHS Doncaster CCG - NHS East Riding of Yorkshire CCG - NHS Greater Huddersfield CCG - NHS Hambleton, Richmondshire and Whitby CCG - NHS Harrogate and Rural District CCG - NHS Hull CCG - NHS Leeds CCG - NHS North East Lincolnshire CCG - NHS North Kirklees CCG - NHS North Lincolnshire CCG - NHS Rotherham CCG - NHS Scarborough and Ryedale CCG - NHS Sheffield CCG - NHS Vale of York CCG - NHS Wakefield CCG | **West Midlands**   - NHS Birmingham and Solihull CCG - NHS Cannock Chase CCG - NHS Coventry and Rugby CCG - NHS Dudley CCG - NHS East Staffordshire CCG - NHS Herefordshire CCG - NHS North Staffordshire CCG - NHS Redditch and Bromsgrove CCG - NHS Sandwell and West Birmingham CCG - NHS Shropshire CCG - NHS South East Staffordshire and Seisdon Peninsula CCG - NHS South Warwickshire CCG - NHS South Worcestershire CCG - NHS Stafford and Surrounds CCG - NHS Stoke On Trent CCG - NHS Telford and Wrekin CCG - NHS Walsall CCG - NHS Warwickshire North CCG - NHS Wolverhampton CCG - NHS Wyre Forest CCG | |
| **Q9. In some cases, we would like to clarify survey responses. If you are happy to be contacted, please provide your contact details below.** *This information will be stored separately from the survey response and you will not be personally identified from any publication* | |  |
| **Name:**  **Preferred method of contact**  **Email:**  **Telephone:**  **Other contact details:** | |  |
| **Q10. Future Correspondence** | |  |
| - I wish to be informed of the study findings. (please provide contact details) - I do not wish to be contacted for future studies. | |  |

##### Please use the freepost envelope provided to return the survey to the researchers at the University of Nottingham

##### Thank you very much for your support of this research!
