# Supplementary material for: Specialist recommendation for chemoprevention medications in patients at familial risk of breast cancer: a cross-sectional survey in England
Source: J Community Genet. 2020 Oct 28;12(1):111–20. doi: 10.1007/s12687-020-00490-4 (PMC7846641; doi:10.1007/s12687-020-00490-4)
Supplement: Supplementary file 2 — (DOCX 22 kb) [file 12687_2020_490_MOESM2_ESM.docx]

**Supplementary Material 2: Unadjusted odds ratio for recommendation of chemoprevention by specialty and by health care role**

| **Responses by specialty** | **Recommendation of chemoprevention by participating services, n (%)** | | **Unadjusted OR (95% CI)** |
| --- | --- | --- | --- |
|  | **Yes** | **No** |  |
| ***HIGH risk, n = 50*** |  |  |  |
| Breast | 31 (78) | 9 (23) | Ref |
| Genetics | 9 (90) | 1 (10) | 2.61 (0.29 – 23.47) |
|  |  |  |  |
| ***MODERATE* risk, n = 49** |  |  |  |
| Breast | 23 (59) | 16 (41) | Ref |
| Genetics | 8 (80) | 2 (20) | 2.78 (0.52 – 14.87) |
|  |  |  |  |
| **Responses by health care role** |  |  |  |
| ***HIGH risk, n = 50*** |  |  |  |
| Nurse & Others* | 14 (67) | 7 (33) | Ref |
| Doctor | 26 (90) | 3 (10) | 4.33 (0.97 – 19.43) |
|  |  |  |  |
| ***MODERATE* risk, n = 49** |  |  |  |
| Nurse & Others* | 11 (55) | 9 (45) | Ref |
| Doctor | 20 (69) | 9 (31) | 1.82 (0.56 – 5.92) |
|  |  |  |  |

*Others include clinical manager and radiographer

CI = confidence interval, OR = odds ratio
